# Impact of *IDH1* and *IDH2* mutational subgroups in AML patients after allogeneic stem cell transplantation

**DOI:** 10.1186/s13045-022-01339-8

**Published:** 2022-09-05

**Authors:** Desiree Kunadt, Sebastian Stasik, Klaus H. Metzeler, Christoph Röllig, Christoph Schliemann, Philipp A. Greif, Karsten Spiekermann, Maja Rothenberg-Thurley, Utz Krug, Jan Braess, Alwin Krämer, Andreas Hochhaus, Sebastian Scholl, Inken Hilgendorf, Tim H. Brümmendorf, Edgar Jost, Björn Steffen, Gesine Bug, Hermann Einsele, Dennis Görlich, Cristina Sauerland, Kerstin Schäfer-Eckart, Stefan W. Krause, Mathias Hänel, Maher Hanoun, Martin Kaufmann, Bernhard Wörmann, Michael Kramer, Katja Sockel, Katharina Egger-Heidrich, Tobias Herold, Gerhard Ehninger, Andreas Burchert, Uwe Platzbecker, Wolfgang E. Berdel, Carsten Müller-Tidow, Wolfgang Hiddemann, Hubert Serve, Matthias Stelljes, Claudia D. Baldus, Andreas Neubauer, Johannes Schetelig, Christian Thiede, Martin Bornhäuser, Jan M. Middeke, Friedrich Stölzel, the A. M. L. Cooperative Group (AMLCG), Study Alliance Leukemia (SAL)

**Affiliations:** 1 Medizinische Klinik und Poliklinik I, Universitätsklinikum Carl Gustav Carus, Dresden, Germany; 2 Laboratory for Leukemia Diagnostics, Department of Medicine III, University Hospital, LMU Munich, Munich, Germany; 3 Klinik und Poliklinik für Hämatologie, Zelltherapie und Hämostaseologie, Universitätsklinikum Leipzig, Leipzig, Germany; 4 Medizinische Klinik A, Universitätsklinikum Münster, Münster, Germany; 5 Medizinische Klinik III, Klinikum Leverkusen, Leverkusen, Germany; 6 Krankenhaus Barmherzige Brüder Regensburg, Regensburg, Germany; 7 Medizinische Klinik Und Poliklinik, Abteilung Innere Medizin V, Universität Heidelberg, Heidelberg, Germany; 8 Klinik für Innere Medizin II, Universitätsklinikum Jena, Jena, Germany; 9 Medizinische Klinik IV, Uniklinik RWTH Aachen, Aachen, Germany; 10 Medizinische Klinik 2, Hämatologie/Onkologie, Goethe-Universität, Frankfurt am Main, Germany; 11 Medizinische Klinik und Poliklinik II, Universitätsklinikum Würzburg, Würzburg, Germany; 12 Institut für Biometrie und Klinische Forschung, Universität Münster, Münster, Germany; 13 Klinik für Innere Medizin 5, Klinikum Nürnberg, Paracelsus Medizinische Privatuniversität, Nuremberg, Germany; 14 Medizinische Klinik 5, Universitätsklinikum Erlangen, Erlangen, Germany; 15 Medizinische Klinik III, Klinikum Chemnitz, Chemnitz, Germany; 16 Klinik für Hämatologie, Universitätsklinikum Essen, Essen, Germany; 17 Abteilung für Hämatologie, Onkologie und Palliativmedizin, Robert-Bosch-Krankenhaus, Stuttgart, Germany; 18 Klinik für Innere Medizin, Schwerpunkt Hämatologie, Onkologie und Immunologie, Philipps Universität Marburg, Marburg, Germany; 19 Klinik für Innere Medizin II, Universitätsklinikum Schleswig-Holstein, Kiel, Germany; 20 DKMS Clinical Trials Unit, Dresden, Germany; 21 National Center for Tumor Diseases, Dresden (NCT/UCC), Dresden, Germany; 22 German Consortium for Translational Cancer Research (DKTK), DKFZ, Heidelberg, Germany

**Keywords:** Acute myeloid leukemia, *IDH* mutations, Allogeneic hematopoietic cell transplantation

## Abstract

**Background:**

The role of allogeneic hematopoietic cell transplantation (alloHCT) in acute myeloid leukemia (AML) with mutated *IDH1/2* has not been defined. Therefore, we analyzed a large cohort of 3234 AML patients in first complete remission (CR1) undergoing alloHCT or conventional chemo-consolidation and investigated outcome in respect to *IDH1/2* mutational subgroups (*IDH1* R132C, R132H and *IDH2* R140Q, R172K).

**Methods:**

Genomic DNA was extracted from bone marrow or peripheral blood samples at diagnosis and analyzed for *IDH* mutations with denaturing high-performance liquid chromatography, Sanger sequencing and targeted myeloid panel next-generation sequencing, respectively. Statistical as-treated analyses were performed using R and standard statistical methods (Kruskal–Wallis test for continuous variables, Chi-square test for categorical variables, Cox regression for univariate and multivariable models), incorporating alloHCT as a time-dependent covariate.

**Results:**

Among 3234 patients achieving CR1, 7.8% harbored *IDH1* mutations (36% R132C and 47% R132H) and 10.9% carried *IDH2* mutations (77% R140Q and 19% R172K). 852 patients underwent alloHCT in CR1. Within the alloHCT group, 6.2% had an *IDH1* mutation (43.4% R132C and 41.4% R132H) and 10% were characterized by an *IDH2* mutation (71.8% R140Q and 24.7% R172K). Variants *IDH1* R132C and *IDH2* R172K showed a significant benefit from alloHCT for OS (*p* = .017 and *p* = .049) and RFS (HR = 0.42, *p* = .048 and *p* = .009) compared with chemotherapy only. AlloHCT in *IDH2* R140Q mutated AML resulted in longer RFS (HR = 0.4, *p* = .002).

**Conclusion:**

In this large as-treated analysis, we showed that alloHCT is able to overcome the negative prognostic impact of certain *IDH* mutational subclasses in first-line consolidation treatment and could pending prognostic validation, provide prognostic value for AML risk stratification and therapeutic decision making.

**Supplementary Information:**

The online version contains supplementary material available at 10.1186/s13045-022-01339-8.

## Background

Isocitrate dehydrogenase (*IDH*) gene mutations are among the most common genetic alterations in acute myeloid leukemia (AML), detected in 15–20% of patients with AML [1, 2]. They represent mutational alterations in early leukemogenesis [3]. Still, their prognostic and predictive relevance is not fully resolved and standard AML risk stratification does not yet include *IDH1* or *IDH2* mutations [4]. However, there is growing evidence that *IDH* mutations contribute both prognostic and predictive value [2, 5, 6]. There have been inconsistent results regarding outcome, including complete remission (CR) rate, relapse-free survival (RFS) and overall survival (OS) depending on *IDH1* and *IDH2* mutational status, respectively [2, 7–12]. For example, some reports attribute a favorable prognosis to *IDH* mutations [8, 13], whereas other reports indicate an adverse prognosis for patients with *IDH* mutations [2, 10, 14–16]. Furthermore, some data suggest the existence of *IDH* mutations have no impact on survival [11, 12]. Supposedly, this is based on different biologic features of certain subtypes of mutations and co-mutational patterns.

To date, two isoforms of *IDH* are known to be potentially mutated in AML encoded on chromosome 2 band q33 (*IDH1*) and chromosome 15 band q26 (*IDH2*), respectively [15, 17, 18]. IDH1 is localized in the cytoplasm and IDH2 is found in mitochondria [19]. Their physiologic role is the enzymatic involvement in the citrate metabolism (*Krebs cycle*) catalyzing decarboxylation of isocitrate to α-ketoglutarate (α-KG) in an NADP+ associated manner. *IDH* mutations induce the loss of this catalytic activity, leading to reduction of α-KG and to the production of the oncometabolite 2-hydroyglutarate (2-HG) accumulating in leukemic cells [20, 21]. 2-HG potentially alters gene expression via DNA and histone hypermethylation and hereby blocks differentiation of hematopoietic progenitor cells [7, 22].

During the last decade, certain mutational subtypes including hotspot mutations affecting codon 132 of *IDH1*, as well as codon R140 and R172 of *IDH2* were identified and have been associated with differential enzymatic potential, consequently suggesting these variants to contribute to disease heterogeneity as well as to contradictions in prognostic predictions [19, 23, 24].

From a therapeutic point of view, they represent attractive drugable targets in clinical routine, as *IDH* inhibitors (e.g., ivosidenib for *IDH1* mutations and enasidenib for *IDH2* mutations) have been introduced for patients with relapsed or refractory AML (r/r AML) and/or elderly/frail AML patients as firstline therapy harboring *IDH* mutations with promising results regarding response and survival [25–27]. Recent reports also demonstrated promising results with the combination of *IDH* inhibitors and hypomethylating agents as frontline therapy [28, 29]. Further, *IDH* inhibitors are investigated in prospective clinical phase I and II trials as maintenance therapy after allogeneic hematopoietic cell transplantation (alloHCT) and/or salvage strategies in case of relapse in the posttransplant setting (e.g., NCT03564821 and NCT04522895).

So far, the role of alloHCT for *IDH* mutated (*IDH*^mut^) AML patients is based on reports from studies with rather small patient numbers or from monocentric analyses [30, 31]. The aim of this study was to evaluate the predictive impact of defined *IDH* mutational subgroups on outcome of alloHCT in first complete remission (CR1) after intensive induction therapy in a well-defined, large multi-center cohort of *IDH*^mut^ AML patients.

## Patients and methods

### Patients

For analysis, we studied a cohort that comprised a total of 3234 intensively treated AML patients under 70 years who either underwent alloHCT (*n* = 852) or chemo-consolidation (*n* = 2382) in CR1. Only patients with sufficient material of bone marrow (BM) and/or peripheral blood (PB) samples available were included in this study. Patients were enrolled within the prospective SAL AML registry (NCT03188874) or one of the following clinical trials: AML96 [32], AML2003 [33], AMLCG1999 [34], AML60+ [35], AMLCG2008 [36], and SORAML [37] (Additional file 1: Table S1). Briefly, intensive chemotherapy regimens consisted of anthracyclines combined with cytarabine in standard dosing. Patients were not treated with *IDH1*- or *IDH2*-inhibitors. Treatment response and outcome measures were classified according to standard criteria [4, 38, 39]. All patients gave their written informed consent on analyses of data. The study was approved by the respective ethics committees and conducted in accordance to the Declaration of Helsinki.

### Molecular and cytogenetic analyses

Pre-treatment BM or PB samples were used for genomic DNA isolation. After DNA extraction, samples were screened for *IDH1* and *IDH2* mutations. Samples collected until 2016 were analyzed in a batched fashion, from 2016 onwards, samples were analyzed in real time. AML patients treated within trials of the SAL registry were screened by denaturing high-performance liquid chromatography (DHPLC) as previously described [40]. In case of aberrant DHPLC-chromatograms, samples were analyzed either by Sanger sequencing or by sensitive ultradeep next-generation sequencing (NGS) [41]. Another NGS-based myeloid panel approach (TruSight Myeloid Panel, Illumina, San Diego, CA, USA) focusing on genes frequently mutated in hematopoietic myeloid entities was used for a subset of SAL registry AML patients [42]. Concordant results were obtained in all SAL patient samples when samples were analyzed with both methods. Concordance was analyzed based on a set of 50 samples representing all mutational variants. A custom targeted NGS assay was deployed for patients enrolled in AML-CG trials [43]. Further mutational profiles (e.g., *FLT3* and *NPM1* mutations) were analyzed as described previously [44, 45]. The lower limit of detection was determined with 0.1% for ultradeep NGS and 1–5% for DHPLC and panel NGS.

### Statistical analyses

Statistical as-treated analyses on the impact of different *IDH1* or *IDH2* mutational subclasses were carried out using the free statistical computing environment R (Version 4.0.3). Continuous variables were compared using the Kruskal–Wallis test, while the Chi-square test was used to compare categorical variables between mutational groups. OS is reported for the whole cohort from study entry until date of death and was censored on date of last follow-up, if no death occurred; RFS is reported from date of CR1 until disease relapse or death and was censored on date of last follow-up. CR and survival rates were evaluated according to the current standard ELN criteria [4]. Effects of alloHCT were estimated using Cox regression models with alloHCT modeled as time-dependent covariate. Simon–Makuch plots were applied to visualize survival according to transplant status. To reduce bias toward benefit of alloHCT due to very early deaths of patients, landmarks of three months for OS (estimated time including two courses of induction therapy and scheduling alloHCT) and one month for RFS (anticipated time from CR1 after induction therapy until alloHCT) were implemented. Due to the time-dependent modeling of alloHCT, all patients start in the non-alloHCT group. Therefore, number at risk in the non-alloHCT groups at start of observational period includes also patients transplanted later. Number at risk of the alloHCT groups at time 0 reflects the number of patients at risk that changed from the non-alloHCT group to the alloHCT group until the first event or censoring was observed in that group, but not earlier than the landmark. Cox regression was also applied to identify independent prognostic variables for survival and to estimate univariate and adjusted hazard ratios (HR). Multivariable analysis included alloHCT in CR1, age at diagnosis, ELN risk group, secondary AML, therapy-related AML and ECOG performance status at diagnosis. The significance level was set at 0.05. For interaction analysis, we used multivariate Cox proportional hazard regression to analyze survival with respect to several variables simultaneously and to provide the hazard ratio for each factor. Furthermore, we performed multivariate Cox regression analysis to study the effect of the interaction of alloHCT and the respective *IDH* submutational groups on outcome.

## Results

### Patients’ characteristics

The study cohort consisted of 3234 patients with AML, whereof a total of 852 patients received alloHCT in CR1 after intense induction therapy. Patients carrying an *IDH*^mut^ were significantly older than patients carrying the wildtype allele (*IDH*^WT^) (*p* < 0.001). Compared to *IDH*^WT^ and *IDH1*^mut^, patients with *IDH2*^mut^ were characterized by a significantly lower serum LDH (*p* = 0.012), whereas *IDH1*^mut^ patients showed a median higher count of peripheral blasts compared to *IDH*^WT^ and *IDH2*^mut^ patients (*p* < 0.001) and bone marrow blasts (*p* < 0.001) at diagnosis, respectively. Regarding other laboratory findings, *IDH1*^mut^ and *IDH2*^mut^ patients had comparable platelet counts at diagnosis, which were significantly higher than those found in *IDH*^WT^ patients (< 0.001). The *IDH*^mut^ cohort harbored a significantly lower rate of complex karyotypes (*p* < 0.001), with *IDH1*^mut^ patients being associated with the lowest rate. Also, patients harboring *IDH1* mutations were more likely to be associated with the ELN2017 favorable-risk and less likely associated with the ELN 2017 adverse-risk category (*p* < 0.001), while patients without *IDH* mutations and *IDH2*^mut^ patients showed similar distributions. No differences in gender, AML subtype (de novo AML, secondary AML, therapy-related AML), white blood count or hemoglobin were detected between *IDH*^mut^ and *IDH*^WT^ patients. An overview of relevant results is depicted in Table 1.

**Table 1:** Overview of the study patients’ characteristics

	AML patients analyzed for *IDH* mutations			*P*-value
	n = 3234			
	*IDH*^WT^	*IDH1*^mut^	*IDH2*^mut^	
	n = 2638	n = 253	n = 353	
Age (years), median (IQR)	51 (40–59)	54 (44–62)	55 (47–62)	**< .001**
Sex, *n*/*N* (%)				.845
Female	1312/2638 (49.7)	130/253 (51.4)	179/353 (50.7)	
Male	1326/2638 (50.3)	123/253 (48.6)	174/353 (49.3)	
Disease status, *n*/*N* (%)				.082
De novo	2238/2622 (85.4)	228/252 (90.5)	304/353 (86.1)	
sAML	255/2622 (9.7)	21/252 (8.3)	34/353 (9.6)	
t-AML	129/2622 (4.9)	3/252 (1.2)	15/353 (4.2)	
Hb (mmol/l), median (IQR)	5.71 (4.9–6.7)	5.65 (5.1–6.6)	5.84 (5–6.8)	.215
Platelets (Gpt/L), median (IQR)	51 (28–95)	71 (36–126)	72 (41–147)	**< .001**
WBC (Gpt/L), median (IQR)	14.98 (3.9–49.1)	12.51 (2.6–44.2)	12.6 (2.8–45.3)	.824
Bone marrow blasts (%), median (IQR)	63 (40–80)	73 (54–88)	70 (44–83)	**< .001**
Peripheral blasts (%), median (IQR)	27 (7–63)	50 (15–81)	36 (9–70)	**< .001**
LDH (U/L), median (IQR)	430 (273–760.8)	425.4 (261–762)	368 (236–624)	**.012**
Complex karyotype, *n*/*N* (%)	258/2532 (10.2)	4/235 (1.7)	14/336 (4.2)	**< .001**
ELN risk 2017, *n*/*N* (%)				**< .001**
Favorable	998/2462 (40.5)	117/227 (51.5)	132/332 (39.8)	
Intermediate	886/2462 (36)	89/227 (39.2)	132/332 (39.8)	
Adverse	578/2462 (23.5)	21/227 (9.3)	68/332 (20.5)	
*NPM1* mut, *n*/*N* (%)	840/2621 (32)	149/252 (59.1)	160 (45.3)	**< .001**
*FLT3*-ITD mut, *n*/*N* (%)	629/2630 (23.9)	55/252 (21.8)	82/353 (23.2)	.741
*CEBPA* mut, *n*/*N* (%)	220/2595 (8.5)	3/253 (1.2)	18/351 (5.2)	**< .001**
*IDH1* mut, *n*/*N* (%)	0/2638 (0)	253/253 (100)	10/353 (2.8)	
R132C	–	92/253 (36.4)	1/10 (10)	
R132G	–	17/253 (6.7)	1/10 (10)	
R132H	–	118/253 (46.6)	8/10 (80)	
R132L	–	12/253 (4.7)	–	
R132S	–	14/253 (5.5)	–	
*IDH2* mut, *n*/*N* (%)	0/2638 (0)	10/253 (4)	353/353 (100)	
R140G	–	–	1/351 (0.3)	
R140L	–	–	6/351 (1.7)	
R140Q	–	10/10 (100)	269/351 (76.6)	
R140 W	–	–	4/351 (1.1)	
R172K	–	–	68/351 (19.4)	
R172S	–	–	1/351 (0.3)	
V161L	–	–	1/351 (0.3)	
WT	–	–	1/351 (0.3)	
*IDH1* and *IDH2* mut, *n/N* (%)	0/2638 (0)	10/253 (4)	10/353 (3)	
*IDH* VAF (%), median (IQR)	–	39 (26.2–43.2)	38.1 (31.7–43.6)	.252
alloHCT in CR1, n/N (%)	714/2638 (27.1)	53/253 (20.9)	85/353 (24.1)	.066

*p*-Values indicating parameters that show significant differences are highlighted in bold

## *IDH* mutations and mutational subgroups

In our cohort of AML patients undergoing either alloHCT or chemo-consolidation in CR1, 18.4% (*n* = 596) had an *IDH*^mut^ with a median variant allele frequency (VAF) of 39% (IQR 26.2–43.2) for *IDH1* and 38.1% (IQR 31.7–43.6) for *IDH2*. A total of 7.8% (*n* = 253) had mutated *IDH1*, 10.9% (*n* = 353) had mutated *IDH2*, while 0.3% (*n* = 10) had mutations in both *IDH1* and *IDH2*. The most common *IDH1* mutational subgroups were R132C (36%) and R132H (47%), while R132G, R132L and R132S were present in only few patients (7%, 5% and 6%, respectively). The two most frequent *IDH2* mutations were R140Q (77%) and R172K (19%) with only a minority of patients (4%) carrying R140G, R140L, R140W, R172S, V161L or WT subtypes.

**Fig. 1:**
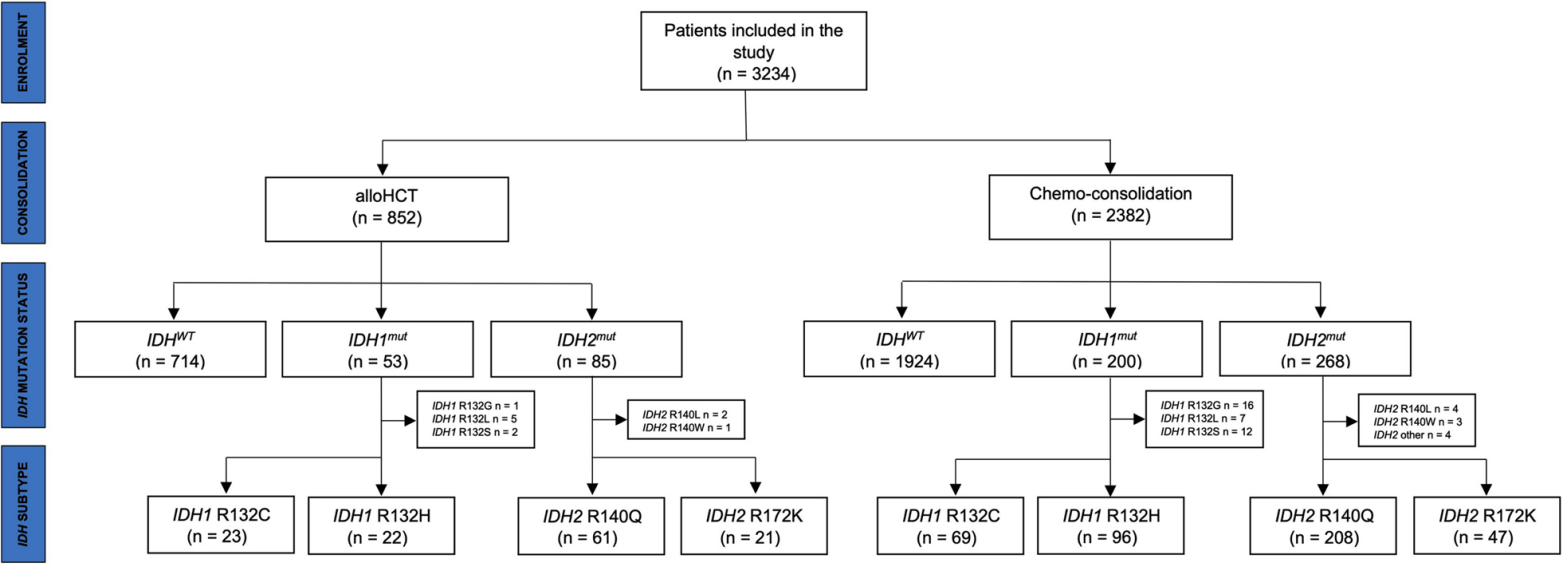
Consort diagram of patients’ distributions. Consort diagram of the study cohorts’ distribution according to the type of consolidation strategy (alloHCT vs. chemo-consolidation), *IDH* mutational status and respective submutational groups

The patients’ distributions were as follows (Fig. 1): Among the 852 patients undergoing alloHCT in CR1, 16.2% (*n* = 138) harbored an *IDH*^mut^. Here, a similar distribution of *IDH*^mut^ could be seen: 6.2% (*n* = 53) harbored an *IDH1* mutation, and again the two major subgroups were R132C (43.4%) and R132H (41.5%) with small numbers of patients mutated in R132G, R132L and R132S (1.9%, 9.4% and 3.8%, respectively). *IDH2* mutations were found in 10% (*n* = 85) of alloHCT patients, also with similar distributions of *IDH2* subgroups R140Q (71.8%) and R172K (24.7%), with a minority of patients carrying R140L (2.4%) and R140W (1.2%). No patients of the alloHCT group had mutations in both *IDH1* and *IDH2*. The non-alloHCT consolidation group included 19.2% (*n* = 458) *IDH*^mut^ patients. Among these patients, 8.4% (*n* = 200) and 11.3% (*n* = 268) carried *IDH1* and *IDH2* mutations, respectively. Only a minority were characterized by mutations in both *IDH1* and *IDH2* (0.4%). In line with the data of the alloHCT group, the two major *IDH1* subgroups in the non-alloHCT cohort were R132C (34.5%) and R132H (48%) and few patients harbored R132G, R132L and R132S (8%, 3.5% and 6%, respectively). Comparing the alloHCT and the non-alloHCT group regarding *IDH* mutational distribution, the alloHCT cohort was characterized by a significant lower percentage of *IDH1* mutations (*p* = 0.042), while there was no statistically differential distribution of *IDH2* mutations between these two groups (*p* = 0.306).

### Co-mutational characteristics

**Fig. 2:**
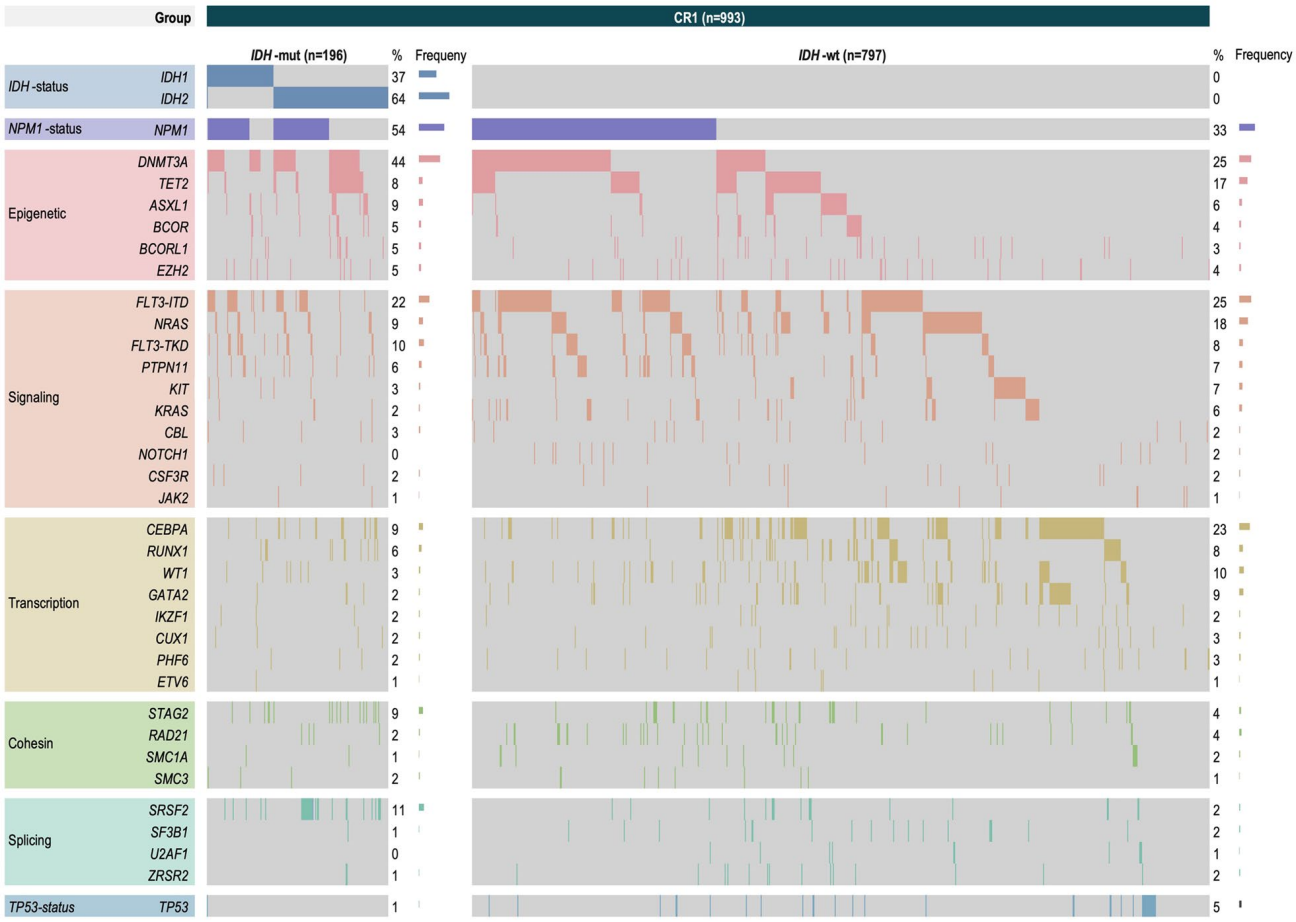
Heatmap of frequent co-mutations according to *IDH* mutation status. Heatmap grouped for epigenetic, signaling, transcription, cohesion and splicing pathways of AML patients achieving CR1 with *IDH* wildtype (*IDH*-wt) or mutated *IDH* (*IDH*-mut). Only patients from the SAL registry with a full dataset of myeloid panel sequencing were included

Regarding co-mutational aspects, the majority of the study cohort had at least two different mutations, with only 3.5% of the *IDH*^WT^ patients, 0.9% of patients carrying an *IDH1*^mut^ and none of the patients with an *IDH2*^mut^ without any co-mutation at all (*p* = 0.012). On the other hand, significant results could be found in the following co-mutational pairs: a significantly higher rate of concomitant *NPM1* mutations was seen in *IDH*^mut^ patients, with *IDH1*^mut^ patients being characterized by the highest rate of co-occurring *NPM1* mutations (*IDH1*^mut^ 59.1% vs. *IDH2*^mut^ 45.3% vs. *IDH*^WT^ 32%, *p* < 0.001). In contrast, the *FLT3*-ITD co-mutational frequency was not significantly different between *IDH*^WT^ and *IDH*^mut^ patients (*p* = 0.741). Despite small number of events, other mutations affecting signaling still showed significant lower rates in the presence of *IDH*^mut^, including mutations in *NRAS* (*IDH1*^mut^ 6.1% vs. *IDH2*^mut^ 5.6% vs. *IDH*^WT^ 12.3%, *p* = 0.006). Biallelic mutations in *CEBPA* were found with a significantly lower frequency in *IDH*^mut^ patients (*IDH1*^mut^ 0.5% vs. *IDH2*^mut^ 1.1% vs. *IDH*^WT^ 6.4%, *p* < 0.001). Further, we detected possible co-mutational patterns with tumor suppressors like *WT1* (*IDH1*^mut^ 1.7% vs. *IDH2*^mut^ 2.2% vs. *IDH*^WT^ 7%, *p* = 0.006). Epigenetic modifiers like mutations in *DNMT3A* and *TET2* were also significantly differentially mutated according to *IDH*^mut^ status (*IDH1*^mut^ 26.1% vs. *IDH2*^mut^ 32.8% vs. *IDH*^WT^ 17.4%, *p* < 0.001 and *IDH1*^mut^ 3.5% vs. *IDH2*^mut^ 7.2% vs. *IDH*^WT^ 12.4%, *p* = 0.003, respectively). Also, mutations in transcription factor *GATA2* and cohesion complex *STAG2* significantly differed between the *IDH*^mut^ and *IDH*^WT^ population (*IDH1*^mut^ 0.9% vs. *IDH2*^mut^ 2.2% vs. *IDH*^WT^ 6.5%, *p* = 0.005 and *IDH1*^mut^ 4.3% vs. *IDH2*^mut^ 6.7% vs. *IDH*^WT^ 2.9%, *p* = 0.029, respectively). An overview of co-mutational distributions is given in Fig. 2 and Table 2.

**Table 2:** Overview of the co-mutational distributions

Mutations	*IDH*^WT^	*IDH1*^mut^	*IDH2*^mut^	*p*-Value
*ASXL1* *n*/*N* (%)	46/1187 (3.9)	5/115 (4.3)	14/180 (7.8)	.059
*BCOR* *n*/*N* (%)	30/1187 (2.5)	3/115 (2.6)	8/180 (4.4)	.342
*BCORL1* *n*/*N* (%)	26/1187 (2.2)	3/115 (2.6)	7/180 (3.9)	.383
*CBL* *n*/*N* (%)	14/1187 (1.2)	2/115 (1.7)	3/180 (1.7)	.779
*CEBPA biallelic (%)*	136/2129 (6.4)	1/195 (0.5)	3/274 (1.1)	**< .001**
*CSF3R* *n*/*N* (%)	13/1187 (1.1)	2/115 (1.7)	2/180 (1.1)	.825
*CUX1* *n*/*N* (%)	22/1187 (1.9)	2/115 (1.7)	2/180 (1.1)	.779
*DNMT3A* *n*/*N* (%)	207/1187 (17.4)	30/115 (26.1)	59/180 (32.8)	**< .001**
*EZH2* n/N (%)	29/1187 (2.4)	5/115 (4.3)	5/180 (2.8)	.472
*GATA2* *n*/*N* (%)	77/1187 (6.5)	1/115 (0.9)	4/180 (2.2)	**.005**
*IKZF1* *n*/*N* (%)	19/1187 (1.6)	2/115 (1.7)	1/180 (0.6)	.543
*JAK2* *n*/*N* (%)	10/1187 (0.8)	0/115 (0)	2/180 (1.1)	.56
*KDM6A* *n*/*N* (%)	5/1187 (0.4)	2/115 (1.7)	0/180 (0)	.089
*KIT* *n*/*N* (%)	54/1187 (4.5)	3/115 (2.6)	2/180 (1.1)	.066
*KRAS* *n*/*N* (%)	48/1187 (4)	1/115 (0.9)	3/180 (1.7)	.075
*NRAS* *n*/*N* (%)	146/1187 (12.3)	7/115 (6.1)	10/180 (5.6)	**.006**
*PHF6* *n*/*N* (%)	23/1187 (1.9)	1/115 (0.9)	2/180 (1.1)	.553
*PTPN11* *n*/*N* (%)	53/1187 (4.5)	6/115 (5.2)	6/180 (3.3)	.711
*RAD21* *n*/*N* (%)	37/1187 (3.1)	0/115 (0)	4/180 (2.2)	.134
*RUNX1* *n*/*N* (%)	61/1187 (5.1)	4/115 (3.5)	10/180 (5.6)	.702
*SMC1A* *n*/*N* (%)	14/1187 (1.2)	1/115 (0.9)	2/180 (0)	.955
*SMC3* *n*/*N* (%)	8/1187 (0.7)	2/115 (1.7)	1/180 (0.6)	.425
*STAG2* *n*/*N* (%)	34/1187 (2.9)	5/115 (4.3)	12/180 (6.7)	**.029**
*TET2* *n*/*N* (%)	147/1187 (12.4)	4/115 (3.5)	13/180 (7.2)	**.003**
*TP53* *n*/*N* (%)	36/1187 (3)	1/115 (0.9)	1/180 (0.6)	.072
*WT1* *n*/*N* (%)	83/1187 (7)	2/115 (1.7)	4/180 (2.2)	**.006**
*ZRSR2* *n*/*N* (%)	13/1187 (1.1)	0/115 (0)	3/180 (1.7)	.399
No co-mutation *n*/*N* (%)	42/1187 (3.5)	1/115 (0.9)	0/180 (0)	**.012**

*p*-Values indicating parameters that show significant differences are highlighted in bold

## Impact of alloHCT on survival according to *IDH* mutational subgroups

**Fig. 3:**
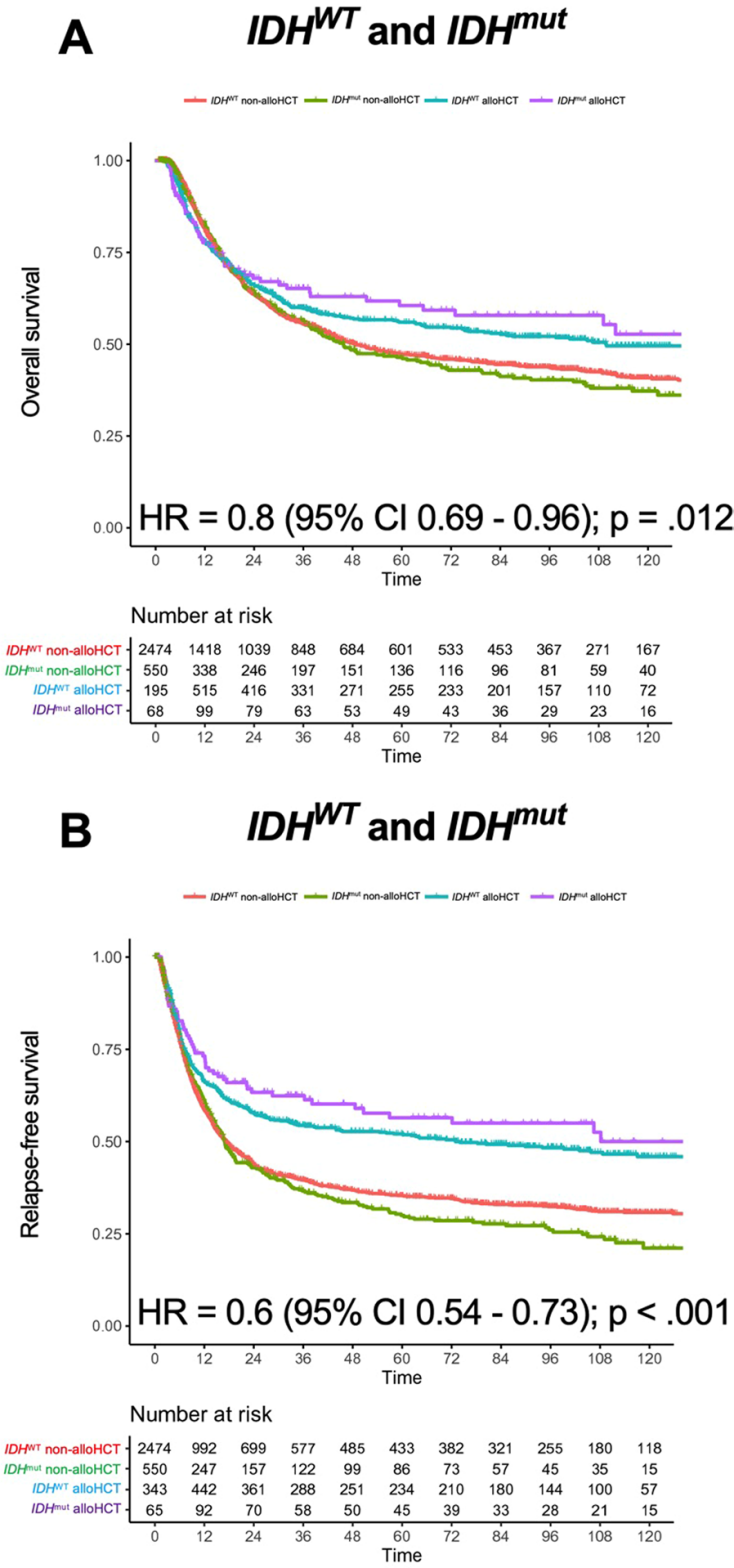
Overall survival and relapse-free survival according to *IDH* mutation status and allogeneic hematopoietic cell transplantation in CR1. Simon–Makuch plots for **a** overall survival and **b** relapse-free survival of AML patients with *IDH* wildtype (WT) or mutated (mut) *IDH* treated with allogeneic hematopoietic cell transplantation (blue for *IDH*^*WT*^ and violet for *IDH*^mut^) or conventional consolidation (red for *IDH*^*WT*^ and green for *IDH*^mut^), respectively; *p*-values were determined with Cox model with time-dependent modeling of alloHCT; time in months

**Fig. 4:**
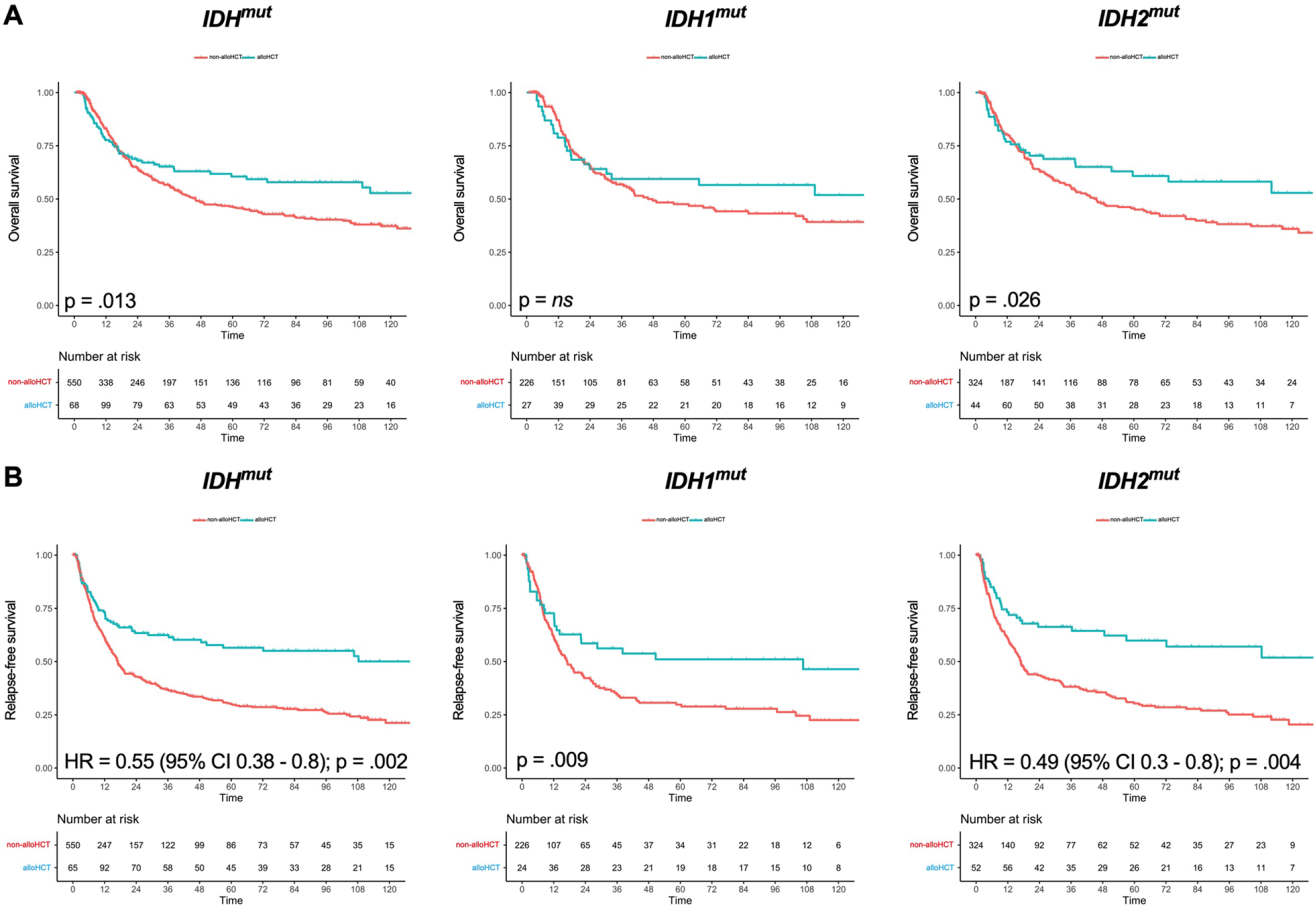
Overall survival according to *IDH*, *IDH1* and *IDH2* mutational status and allogeneic hematopoietic cell transplantation in CR1. Simon–Makuch plots for **a** overall survival and **b** relapse-free survival of AML patients with mutated (mut) *IDH*, *IDH1* and *IDH2* treated with allogeneic hematopoietic cell transplantation (blue) or conventional consolidation (red), respectively; *p*‐values were determined with Cox model with time‐dependent modeling of alloHCT; time in months; ns = not significant

Regarding the whole cohort undergoing alloHCT or conventional chemo-consolidation in CR1, a significant survival benefit for alloHCT in both *IDH*^WT^ and *IDH*^mut^ group was revealed (Fig. 3). This positive effect for alloHCT is valid for OS (HR = 0.8, 95% CI 0.69–0.96, *p* = 0.012; Fig. 3a), as well as RFS (HR = 0.6, 95% CI 0.54–0.73, *p* < 0.001; Fig. 3b). Median OS was 49 months (*IDH*^WT^ non-alloHCT) versus 46 months (*IDH*^mut^ non-alloHCT) versus 110 months (*IDH*^WT^ alloHCT), while the *IDH*^mut^ cohort receiving alloHCT did not reach median OS. Median RFS was 17 months (*IDH*^WT^ non-alloHCT) versus 17 months (*IDH*^mut^ non-alloHCT) vs. 74 months (*IDH*^WT^ alloHCT), while median RFS was also not reached in the *IDH*^mut^ cohort receiving alloHCT. Interestingly, when undergoing alloHCT, a trend toward better OS and RFS could be detected in the *IDH*^mut^ group compared with the *IDH*^WT^ group. Vice versa, a negative trend for survival was revealed in *IDH*^mut^ patients compared with *IDH*^WT^ patients when receiving chemo-consolidation only (Fig. 3). Overall, there was no statistical difference in OS of either consolidation strategy for patients carrying an *IDH1* mutation (5-year OS 40% [non-alloHCT] vs. 47% [alloHCT], *p* = 0.27; Fig. 4a), alloHCT led to a better RFS in univariate analysis (5-year RFS 30% [non-alloHCT] vs. 51%, *p* = 0.009; Fig. 4b). In contrast, *IDH2*^mut^ patients gained an advantage in OS when undergoing alloHCT in univariate analysis (5-year OS 46% [non-alloHCT] vs. 61% [alloHCT], *p* = 0.026; Fig. 4a) and RFS was significantly better for alloHCT in multivariable analysis (5-year RFS 30% [non-alloHCT] vs. 60% [alloHCT]; HR = 0.49, 95% CI 0.3–0.8, *p* = 0.004; Fig. 4b).

**Fig. 5:**
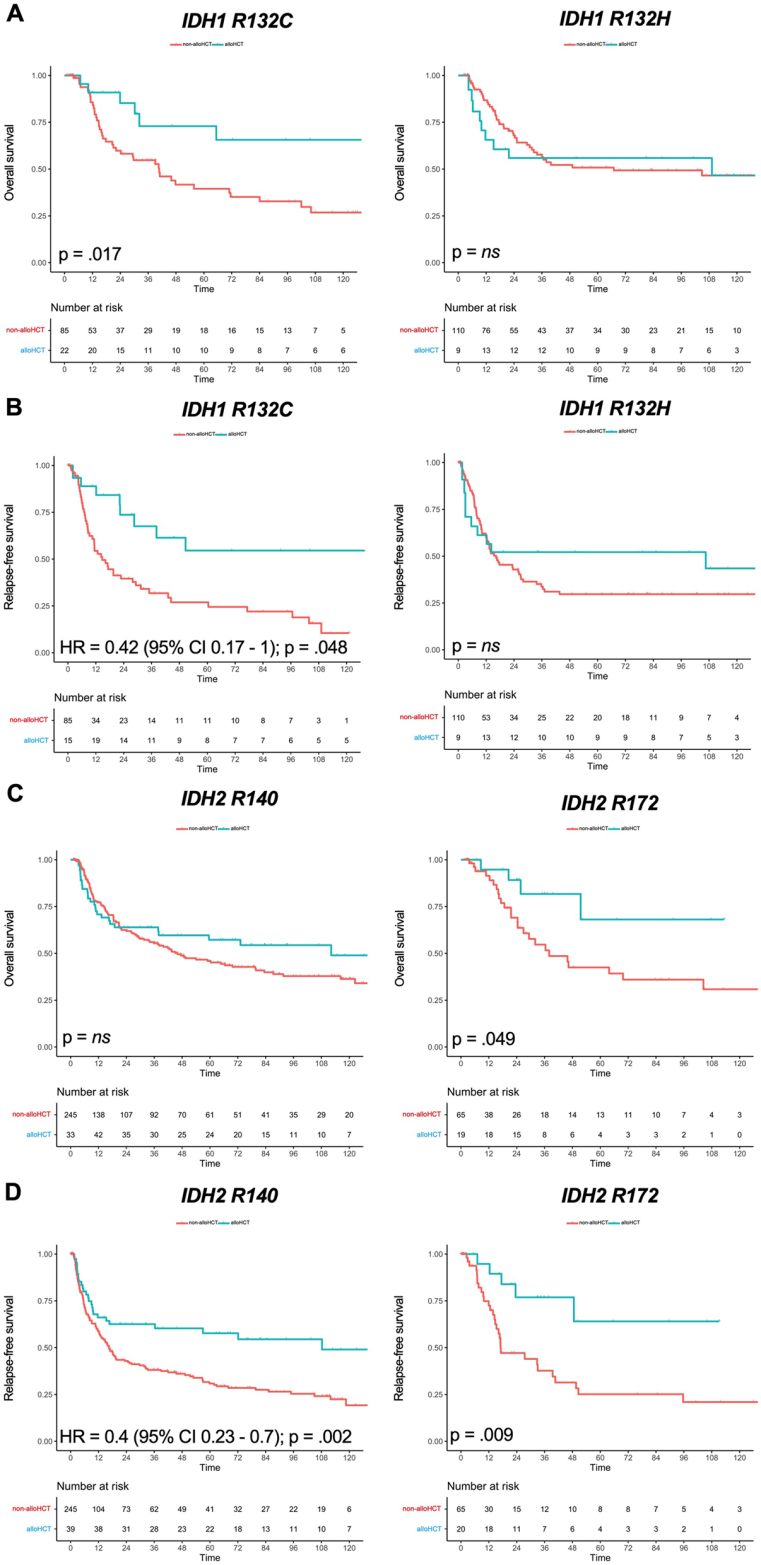
Overall survival and relapse-free survival according to *IDH1* and *IDH2* mutational subgroups and allogeneic hematopoietic cell transplantation in CR1. Simon–Makuch plots for **a** and **c** overall survival and **b** and **d** relapse-free survival of AML patients with mutated *IDH1* R132C, *IDH1* R132H, *IDH2* R140 and *IDH2* R172 mutational subgroups treated with allogeneic hematopoietic cell transplantation (blue) or conventional consolidation (red), respectively; *p*-values were determined with Cox model with time-dependent modeling of alloHCT; time in months; ns = not significant

More importantly, the relevance of mutational subtypes in *IDH1* and *IDH2* on survival could be delineated in our analysis (Fig. 5). Patients with *IDH1* R132C had a higher OS when undergoing alloHCT in univariate analysis (5-year OS 40% [non-alloHCT] vs. 73% [alloHCT], *p* = 0.017; Fig. 5a), which was even more pronounced for RFS in multivariable analysis (5-year RFS 27% [non-alloHCT] vs. 55% [alloHCT]; HR = 0.42, 95% CI 0.17–1, *p* = 0.048; Fig. 5b). However, *IDH1* R132H was not associated with superior survival (Fig. 5a,b). AlloHCT patients carrying *IDH2* variant R140 mutations showed no significant difference in OS regarding the respective consolidation strategy (Fig. 5c), but significantly higher RFS compared with the chemo-consolidation group in multivariable analysis (5-year RFS 31% [non-alloHCT] vs. 58% [alloHCT]; HR = 0.4, 95% CI 0.23–0.7, *p* = 0.002; Fig. 5d). *IDH2* variant R172 was associated with increased OS and RFS when undergoing alloHCT in univariate analysis (5-year OS 43% [non-alloHCT] vs. 68% [alloHCT], *p* = 0.049; Fig. 5c and 5-year RFS 25% [non-alloHCT] vs. 64% [alloHCT]; *p* = 0.009, respectively; Fig. 5d).

### Multivariable analysis

Further multivariable modeling of established factors affecting survival of AML patients (Additional file 1: Fig. S1) revealed significant results regarding age (HR = 1.03, *p* < 0.001), favorable (HR = 0.6, *p* < 0.001) and adverse (HR = 1.7, *p* < 0.001) risk category according to ELN risk stratification and ECOG performance status 0–1 (HR = 0.7, *p* < 0.001) on OS when analyzing the whole cohort. RFS was also significantly influenced by age (HR = 1.02, *p* < 0.001), ELN favorable (HR = 0.6, *p* < 0.001) and adverse (HR = 1.5, *p* < 0.001) and ECOG performance status 0–1 (HR = 0.8, *p* = 0.001). Including *IDH* submutational groups into multivariable analysis, *IDH2* R172 was an independent predictor for better OS (HR = 0.5, *p* = 0.02), which was even more pronounced for RFS (HR = 0.4, *p* < 0.001). *IDH1* mutational subclasses were associated with a trend toward better OS (R132C, HR = 0.86, *p* = 0.5; R132H, HR = 0.89, *p* = 0.6), whereas *IDH2* R140 showed a trend toward inferior OS (HR = 1.1, *p* = 0.35). Similar results were obtained in multivariable analysis for RFS where *IDH1* R132C showed a trend toward better RFS (HR = 0.77, *p* = 0.19) and *IDH2* R140 a trend toward worse RFS (HR = 1.2, *p* = 0.1).

### Interaction analysis

**Fig. 6:**
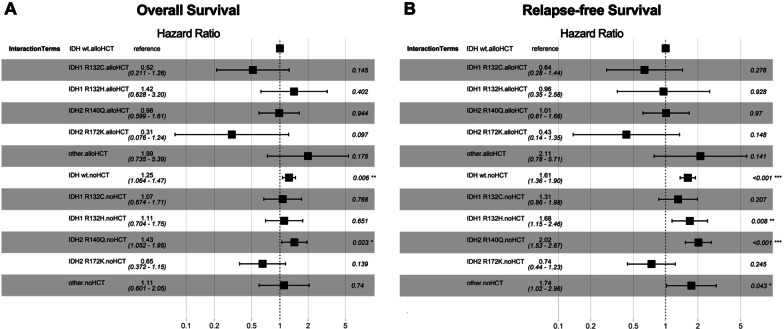
Multivariable Cox model with single interaction terms. Single interaction terms for **a** overall survival and **b** relapse-free survival for *IDH* submutational groups *IDH1* R132C, *IDH1* R132H, *IDH2* R140Q and *IDH2* R172K, other *IDH* mutational subgroups (other) or *IDH* wildtype (wt) with either allogeneic hematopoietic cell transplantation (alloHCT) or chemo-consolidation (noHCT); *p*-values and hazard ratios were determined with Cox model

For studying the effect of the interaction of alloHCT and the respective *IDH* submutational groups on outcome, we performed interaction analysis with the interaction of alloHCT and *IDH*^*WT*^ AML patients as the reference term (Fig. 6a for OS, Fig. 6b for RFS). Interaction analysis demonstrated a trend toward improved outcomes for the interaction of alloHCT and *IDH1* R132C (OS, HR = 0.52, *p* = 0.15; RFS, HR = 0.64, *p* = 0.28) and *IDH2* R172 (OS, HR = 0.31, *p* = 0.1; RFS, HR = 0.43, *p* = 0.15), although they did not reach statistical significance. In contrast, interaction analysis of alloHCT and *IDH1* R132H predicted a trend toward worse OS (HR = 1.42, *p* = 0.4). The interaction of alloHCT and *IDH1* R132H for RFS (HR = 0.96, *p* = 0.93), as well as the interaction of alloHCT and *IDH2* R140Q (OS, HR = 0.98, *p* = 0.94; RFS, HR = 1, *p* = 0.97) predicted similar outcomes like the *IDH*^*WT*^ cohort that was allografted. Other mutational *IDH* subgroups in the alloHCT cohort were almost at double risk for decreased outcome (OS, HR = 1.99, *p* = 0.18; RFS, HR = 2.11, *p* = 0.14). In contrast, the effect of the interaction of *IDH*^*WT*^ and *IDH* mutational subclasses and chemo-consolidation only predicted worse outcome, which was mostly pronounced in the terms of *IDH*^*WT*^ (OS, HR = 1.25, *p* = 0.006; RFS, HR = 1.61, *p* < 0.001), *IDH1* R132H (RFS, HR = 1.68, *p* = 0.008), *IDH2* R140Q (OS, HR = 1.43, *p* = 0.023; RFS, HR = 2.02, *p* < 0.001) and other *IDH* mutational subgroups (RFS, HR = 1.74, *p* = 0.043). However, the interaction term of chemo-consolidation and *IDH2* R172K demonstrated a trend toward improved outcome (OS, HR = 0.65, *p* = 0.139; RFS, HR = 0.74, *p* = 0.245).

## Discussion

Here, we report that the unfavorable prognostic impact of specific *IDH* mutational subgroups on survival can be mitigated by alloHCT as frontline consolidation strategy in a well-defined AML study cohort. To the best of our knowledge, this is the largest multicenter analysis to determine the prognostic effect of *IDH* mutations in the course of alloHCT, comprising a cohort of 852 AML patients transplanted in CR1.

Considering the significant biological and molecular heterogeneity of AML, the ideal consolidation therapy is one of the main foci of scientific and clinical interest. Previous studies generated partly controversial results, either associating *IDH*^mut^ with better outcome [8, 13] and studies reporting a negative impact on outcome [2, 14, 16]. More recently, it was shown that *IDH* mutational subgroups associated with different biological features have different prognostic impact, suggesting to provide an explanation for inconsistent results concerning prognosis and survival so far [6, 23, 46]. To add a next level of complexity, different mutational *IDH* variants are associated with differential co-mutational patterns or karyotypes, incorporating prognostic value and even potentially defining distinct genomic categories in AML [10, 15, 23, 46–49]. As recently shown, considering differential co-mutational rates of epigenetic modifiers like *DNMT3A* and *TET2* in combination with the hypermethylating ability of *IDH*^mut^, some suppose *IDH*^mut^ to be predictive of susceptibility to hypomethylating agents [50, 51]. These results indicate the need for more clarification in the clonal composition, hierarchy and development in the concept of disease biology of *IDH*^mut^ AML, as latest data suggest [52].

In accordance with previous reports, nearly 20% of the patients analyzed were characterized by *IDH*^mut^. Similar to our recent analysis [6], a significantly higher rate of *NPM1*/*IDH* co-mutations was seen. In the presence of *IDH*^mut^, our present analysis also revealed significant differential co-mutational distributions compared with *IDH*^WT^ patients. These patterns, as well as their prognostic impact, have to be considered when analyzing outcomes in AML patients, as our study did not include these co-mutational aspects. Also in line with previous data, our *IDH*^mut^ cohort was characterized by significantly older age, as well as lower LDH concentration (especially for *IDH2*^mut^ patients) and a higher count of peripheral blasts (pronounced in *IDH1*^mut^ patients) and bone marrow blasts [48] .

Most importantly, our present data is demonstrating a beneficial effect of alloHCT for *IDH*^mut^ AML patients, which is in line with recently published data of Duchmann et al*.* who demonstrated superior OS for *IDH*^mut^ AML patients treated with alloHCT in CR1 [46], but also contrary to previous studies associating *IDH* mutations with higher rates of relapse after alloHCT [30]. *IDH*^mut^ patients showed a trend toward prolonged OS and improved RFS compared with their wildtype counterparts when undergoing alloHCT and shorter OS and RFS compared with *IDH*^WT^ patients when receiving chemo-consolidation after CR1. Focusing on *IDH1* mutations, R132C was characterized by an improved OS and RFS if transplanted in CR1, an effect which could not be shown for R132H. This improvement in survival was shown previously only regarding OS and without discriminating between R132 variants [46]. Whether the difference in prognosis implicated by R132H is due to increased 2-HG levels causing blockage of differentiation in hematopoiesis needs further investigation [53]. Interestingly, R132C patients had the worst 5-year OS compared with the other three analyzed subtypes when consolidated with chemotherapy after CR1 in our study, but the highest 5-year OS of all *IDH* subgroups when treated with alloHCT in CR1, begging the question of differential susceptibility to allografting among *IDH* mutational subgroups. Furthermore, when incorporating our recently published data including co-mutational patterns of *IDH*^mut^ patients into our current analysis, we did not see a clear correlation between improved OS and a high frequency of *NPM1* co-mutations, as *IDH1* R132C was the subgroup characterized by the lowest rate of co-occurring *NPM1* mutations among all *IDH1*^mut^ patients (*IDH1* R132C 24.2% vs. *IDH1* R132H 71% vs. *IDH1* other 64.2%) and was also less likely to harbor *NPM1* mutations compared to *IDH*^WT^ patients (28.4%) [6]. The same trend is seen for *FLT3*-ITD, another mutation known to benefit from alloHCT, with *IDH1* R132C characterized by the lowest rate of co-occurring *FLT3*-ITD mutations [6]. On the other hand, *IDH1* R132H, which is associated with the highest rate of co-occurring *NPM1* mutations (71% of patients) according to our recently published data, demonstrates the worst 5-year OS when undergoing transplantation. These retrospective data suggest that *IDH1* R132C could be a clear profiteer from alloHCT, as our recent analysis also revealed a trend toward reduced OS in patients carrying *IDH1* variant R132C after intensive induction chemotherapy, and that there could be a beneficial aspect of alloHCT alone independent of *NPM1* or *FLT3*-ITD mutation status, providing a chance to overcome the worse prognosis for patients lacking “favorable” mutations like *NPM1*. However, low patient numbers in these subgroups of our analysis need to be taken into account and further validation is needed.

Patients with *IDH2* subtype R140 had no differential OS probability, but significantly prolonged RFS after alloHCT in CR1. In contrast, *IDH2* R172 was characterized by significant higher OS, as well as higher RFS in the alloHCT cohort. These data suggest that allografting AML patients with an *IDH2* R172 mutation as consolidation strategy is a considerable option for these patients. Recently, Linch et al. also reported improved survival of AML patients carrying *IDH2* R172 variant compared with a historical *IDH2* R172 cohort presenting with poor prognosis, relating increased use of alloHCT as consolidation after CR1 with longer OS in the later cohort, as induction strategy was almost unchanged and patients of the later cohort were even significantly older [54]. Additionally, high levels of 2-HG as an oncometabolite and prognostic indicator are paralleled by unfavorable outcome and R172 has been shown to induce higher levels of 2-HG than R140 [24, 55–57]. However, our present data reveal an independent beneficial prognostic impact on survival of *IDH2* R172. Again, although our *IDH2* cohort was bigger and provided more statistical power, small patient numbers and underlying co-mutational patterns have to be considered when interpreting these data, although *IDH2* R172 seems to define a distinct genetic AML subgroup, being mutually exclusive from class-defining genetic aberrations like *NPM1* mutations as reported previously [6, 23, 49]. Duchmann and colleagues recently attributed co-occurring *NPM1* mutations in *IDH1* and *IDH2* R140-mutated patients as the main prognostic component for improved survival [46]. However, these results were not analyzed in patients undergoing alloHCT or only in a small transplant cohort, respectively. In our non-alloHCT cohort, we could evaluate corresponding results when incorporating our recent results on *IDH* mutations and co-mutations [6]. Briefly, *IDH* subtypes with the highest 5-year OS in our present analysis (e.g., *IDH1* R132H with 51% and *IDH2* R140 with 46%) were also the subgroups with the highest frequencies of co-occurring *NPM1* mutations (*IDH1* R132H with 71% and *IDH2* R140 with 49% of patients carrying additional *NPM1* mutations). Along with these results, the *IDH* subgroup with a lower rate *NPM1* mutation (*IDH1* R132C with 24%) had the worst 5-year OS in our non-alloHCT cohort (40%). Again, *IDH2* R172 was characterized by improved prognosis (5-year OS of 68%) independent of *NPM1* mutations (with 2% of patients carrying *NPM1*) [6]. Hence, our results are in line with the data Duchmann et al., with an implied association that seems to arise between improved survival and *NPM1* mutation status.

In summary, a better survival for AML patients with mutated *IDH* undergoing alloHCT in CR1 could be illustrated, with modest to statistically significant differences depending on the underlying *IDH1/2* mutational variant. The improved prognostic effect of alloHCT was mostly pronounced in the mutational subgroups *IDH1* R132C and *IDH2* R172. However, limitations of this retrospective analysis include the lack of information about donor availability, patients’ performance status after induction therapy and small patient numbers for subgroup analysis. Still, the compiled results highlight the urgent need for increased knowledge about disease biology and the relevance of prognostic and predictive markers in order to apply individually adjusted treatment decisions and optimized consolidation strategies in AML. Ongoing studies are currently investigating the implementation of *IDH* inhibitors in the frontline setting of induction therapy (NCT03839771 and NCT04493164), which will add valuable data for the re-evaluation of the role of alloHCT in *IDH*^mut^ patients when pre-treated with *IDH* inhibitors during induction, consolidation or as a maintenance therapy after alloHCT.

## Conclusion

On the basis of our results, it is arguable that defined *IDH* mutational subgroups introduce predictive and prognostic potential in different therapeutic settings. Furthermore, the differential responsiveness and “alloreactivity” of single *IDH* subclasses to alloHCT in CR1 should initiate further prospective investigations to validate these findings, especially in respect of co-mutational patterns influencing the predictive value of *IDH* mutations, offering the chance to add information for refined AML risk classifications to improve survival for AML patients.

## Supplementary Information

**Additional file 1: Table S1** Overview of the clinical trials the study patients were selected from. **Figure S1** Forrest Plot of variables evaluated in univariate analysis. Multivariate Cox proportional hazard regression for (**A**) overall survival and (**B**) relapse-free survival.

## Data Availability

The datasets used and/or analyzed during the current study are available from the corresponding author on reasonable request.

## Abbreviations

2-HG: 2-Hydroyglutarate
α-KG: α-Ketoglutarate
alloHCT: Allogeneic hematopoietic cell transplantation
AML: Acute myeloid leukemia
AML-CG: AML-Cooperative Group
BM: Bone marrow
*CEBPA*: CCAAT/enhancer-binding protein alpha
CI: Confidence interval
CR1: First complete remission
DHPLC: Denaturing high-performance liquid chromatography
*DNMT3A*: DNA (cytosine-5)-methyltransferase 3A
ECOG: Eastern Cooperative Oncology Group
ELN: European Leukemia Net
Fig.: Figure
*FLT3*: Fms-like tyrosine kinase 3
*GATA2*: GATA-binding factor 2
HR: Hazard ratio
*IDH*: Isocitrate dehydrogenase
*IDH*^mut^: *IDH* Mutated
*IDH*^WT^: *IDH* Wildtype
LDH: Lactate dehydrogenase
NCT: National clinical trial
NGS: Next-generation sequencing
*NPM1*: Nucleophosmin 1
*NRAS*: Neuroblastoma RAS
OS: Overall survival
PB: Peripheral blood
RFS: Relapse-free survival
r/r: Relapsed/refractory
SAL: Study Alliance Leukemia
*STAG2*: Stromal Antigen 2
*TET2*: Tet methylcytosine dioxygenase 2
VAF: Variant allele frequency
vs.: Versus
*WT1*: Wilms Tumor 1
