# Supplementary material for: Impact of IDH1 and IDH2 mutational subgroups in AML patients after allogeneic stem cell transplantation
Source: J Hematol Oncol. 2022 Sep 5;15:126. doi: 10.1186/s13045-022-01339-8 (PMC9442956; doi:10.1186/s13045-022-01339-8)
Supplement: Supplementary file 1 — Additional file 1: Table S1 Overview of the clinical trials the study patients were selected from. Figure S1 Forrest Plot of variables evaluated in univariate analysis. Multivariate Cox proportional hazard regression for (A) overall survival and (B) relapse-free survival. [file 13045_2022_1339_MOESM1_ESM.docx]

**SUPPLEMENT**

**Suppl. Table 1**

| **Clinical trial** | **National Clinical Trial**  **identifier** | **Investigational key points** | **Remission Assessment** |
| --- | --- | --- | --- |
| ***Study Alliance Leukemia* (SAL) registry** | NCT03188874  (2010 - ongoing) | - Prospective registry of adult patients with AML: clinical registry and biomaterial database | Until 2017:  ELN 2010  Döhner *et al.*  Blood. 2010  From 2017 – ongoing:  ELN2017  Döhner *et al.*  Blood. 2017 |
| **AML96** | NCT00180115  (1996-2008) | - risk-adapted postremission treatment strategy for high-risk AML patients: related and unrelated allogeneic stem cell transplantation - risk-adapted postremission treatment strategy for standard-risk AML patients: related allogeneic and autologous stem cell transplantation - first postremission-course: intermediate-dose cytarabine (12 g/m^2^, I-MAC) vs. high-dose cytarabine (36 g/m^2^, H-MAC) combined with mitoxantrone | Cheson BD *et al.* *J Clin Oncol.* 2003 |
| **AML2003** | NCT00180102  (2003-2009) | - value of early allogeneic stem cell transplantation in aplasia after induction therapy for high-risk AML patients - relevance of autologous transplantation and the benefit of additional substances within postremission therapy: m-AMSA or mitoxantrone - up-front randomization in four therapy arms with two cross-classifying factors of two stages: intensified vs. standard therapy and cytarabine (18 g/m^2^) vs. cytarabine (12 g/m^2^) + mitoxantrone (30 g/m^2^) + amsacrine (500 mg/m^2^) | Cheson BD *et al.* *J Clin Oncol.* 2003 |
| **AMLCG1999** | NCT00266136  (1999 – 2012) | - Induction: TAD-HAM vs. HAM-HAM - TAD: cytarabine (100 mg/m^2^ days 1 and 2 and bid on days 3 to 8) + daunorubicin (60 mg/m^2^ days 3, 4, 5) + thioguanine (100 mg/m^2^ bid days 3 to 9) - HAM: cytarabine (3 g/m^2^ (patients younger than 60 years) or 1 g/m^2^ (patients ≥ 60 years) bid days 1 to 3) + mitoxantrone (10 mg/m^2^ days 3 to 5) - Consolidation: one course TAD - Maintenance: monthly cytarabine (100 mg/m^2^ bid days 1 to 5) + daunorubicin (45 mg/m^2^ days 3 and 4) + thioguanine (100 mg/m^2^ bid days 1 to 5) vs. cyclophosphamide (1 g/m^2^ day 3) with the second agent changing in a rotating sequence - G-CSF priming vs. no priming before allogeneic stem cell transplantation - Autologous stem cell transplantation - Allogeneic stem cell transplantation | Cheson BD *et al.* *J Clin Oncol.* 2003  Döhner *et al.*  *Blood. 2010* |
| **AML60+** | NCT00180167  (2005 – 2010) | - Mitoxantron (10mg/m^2^) for 3 days + cytarabine (1g/m^2^) bid on days 1+3+5+7 vs. DA 7+3 (45mg/m^2^ daunorubicin) | Cheson BD *et al.* *J Clin Oncol.* 2003 |
| **AMLCG2008** | NCT01382147  (2009 – 2017) | - S-HAM vs. TAD-HAM (younger) or HAM-HAM (elderly) - TAD: cytarabine 100 mg/m^2^ days 1 and 2 and bid on days 3 to 8 + daunorubicin 60 mg/m^2^ days 3, 4, 5 + thioguanine 100 mg/m^2^ bid days 3 to 9 - HAM: cytarabine 3 g/m^2^ (patients younger than 60 years) or 1 g/m^2^ (patients ≥ 60 years) bid days 1 to 3 + mitoxantrone 10 mg/m^2^ days 3 to 5 - S-HAM:  cytarabine (3 g/m^2^) bid days 1 to 2 and days 8 to 9 (1 g/m^2^ in patients ≥ 60 years) + mitoxantrone (10 mg/m^2^) days 3 to 4 and days 10 to 11 | Cheson BD *et al.* *J Clin Oncol.* 2003 |
| **SORAML** | NCT00893373  (2011-2014) | - Standard therapy + Sorafenib 2 x 400 mg/d vs. standard therapy + placebo | Cheson BD *et al.* *J Clin Oncol.* 2003 |

**Suppl. Table 1 Overview of the clinical trials the study patients were selected from**

**Suppl. Figure 1**

**
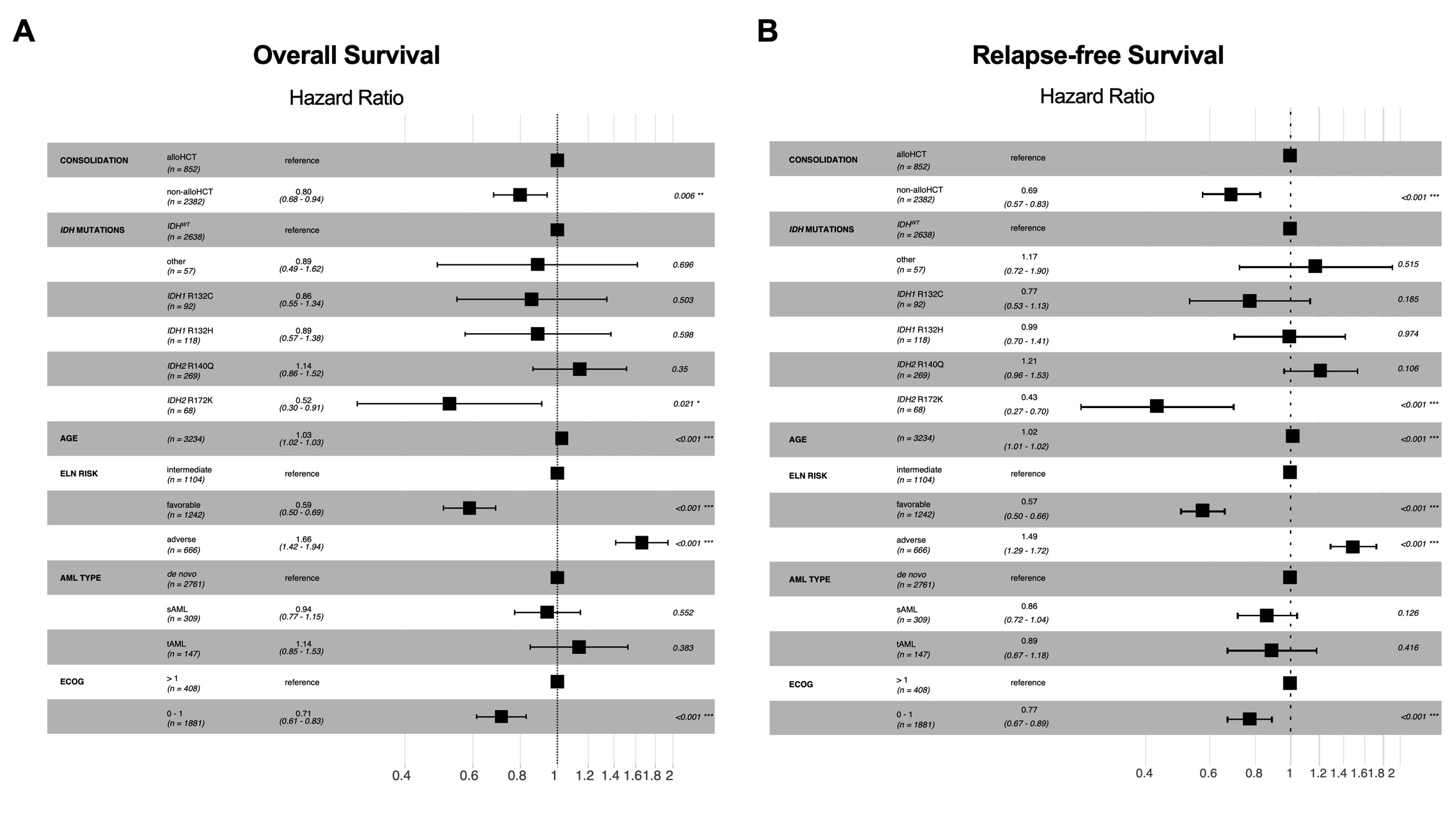
**

**Suppl. Figure 1 Forrest Plot of variables evaluated in univariate analysis**

Multivariate Cox proportional hazard regression for **(A)** overall survival and **(B)** relapse-free survival.
